# BHLHE41–SLC7A11 transcriptional axis and chromatin remodeling signatures in osteogenic-lineage disulfidptosis-like stress in osteoporosis

**DOI:** 10.3389/fgene.2026.1794298

**Published:** 2026-04-02

**Authors:** Xiaoming Zhao, Wenya Xue, Jun Gao, Yilei Zhang, Jinghong Chen, Yingang Zhang, Song Long

**Affiliations:** 1 Department of Orthopedics, The First Affiliated Hospital, Xi’an Jiaotong University, Xi’an, Shaanxi, China; 2 Department of Orthopedics, Xi’an Fengcheng Hospital, Xi’an, Shaanxi, China; 3 The Institute of Molecular and Translational Medicine, Department of Biochemistry and Molecular Biology, School of Basic Medical Sciences, Xi’an Jiaotong University Health Science Center, Xi’an, Shaanxi, China; 4 Institute of Endemic Diseases, School of Public Health, Xi’an Jiaotong University Health Science Center, National Health Commission (NHC) Key Laboratory of Environment and Endemic Diseases, Xi’an, Shaanxi, China; 5 Department of Pharmacy, Honghe Hospital Affiliated to Kunming Medical University, Honghe, Yunnan, China

**Keywords:** osteoporosis, bone microenvironment, disulfidptosis, SLC7A11, BHLHE41

## Abstract

**Background:**

Osteoporosis (OP) is characterized by impaired bone homeostasis in which bone resorption exceeds bone formation. Disulfidptosis, a recently described disulfide stress–induced cell death program linked to cytoskeletal collapse, has been suggested to contribute to OP, yet its cell-type–specific relevance within the bone marrow mesenchymal stem cell (BM-MSC) osteogenic lineage—and the upstream upstream transcriptional and epigenetic programs shaping this stress response—remain unclear.

**Methods:**

This study integrated a peripheral blood monocyte microarray dataset (GSE56815; 20 OP vs. 20 controls) with single-cell RNA sequencing to characterize disulfidptosis-related programs in OP. OP-associated disulfidptosis genes and molecular subtypes were identified using co-expression and differential analyses. Disulfidptosis score and upstream regulators across bone marrow cell populations were inferred from single-cell data using regulon analysis with motif/cis-regulatory evidence. Chromatin remodeling–related gene modules in osteoblasts were additionally scored to assess epigenetic activation/repression programs and their association with BHLHE41.

**Results:**

Integrated WGCNA with gene-overlap screening pinpointed 17 OP-linked disulfidptosis signature genes, highlighted by a marked increase of SLC7A11, and unsupervised stratification separated OP into two molecular subtypes. Single-cell analyses showed that disulfidptosis score was enriched in the osteoblast-like subset of BM-MSCs, implicating osteoblasts as a major affected population. Network inference further nominated BHLHE41 as a potential upstream driver of SLC7A11 and connected its expression with a disulfidptosis-tolerant phenotype in OP-derived BM-MSCs. Chromatin remodeling pathway scoring indicated altered epigenetic state programs in OP osteoblasts, and SIRT1 was preferentially upregulated in BHLHE41-high osteoblasts.

**Conclusion:**

This study provides a cell-type–resolved map of disulfidptosis-related stress in osteoporosis by integrating bulk and single-cell transcriptomics. We propose a BM-MSC osteogenic-lineage–associated BHLHE41–SLC7A11 axis and link it to chromatin remodeling signatures in osteoblasts, offering a rationale for precision strategies targeting disulfide-stress vulnerability in OP.

## 1 Introduction

Osteoporosis (OP) is a systemic metabolic disorder of the skeleton that is marked by decreased bone mass together with deterioration of bone microstructure (Feng et al., 2024). At its core, OP arises from disrupted bone homeostasis, in which osteoclast-driven resorption continually outpaces the bone-forming capacity of osteoblasts (Song et al., 2022). Such a persistent remodeling imbalance ultimately increases bone fragility and markedly heightens the likelihood of fractures (Gopinath, 2023). As worldwide population aging continues to accelerate, osteoporosis has increasingly become a major public health burden (Rashki Kemmak et al., 2020). In current clinical practice, antiresorptive agents—including bisphosphonates (Rizzoli and Ferrari, 2005) and selective estrogen receptor modulators (Caires et al., 2017)—can suppress bone resorption effectively, yet their shortcomings remain evident (Fu et al., 2025). Specifically, these therapies are generally insufficient to robustly stimulate new bone formation or truly restore lost bone, and they may also induce gastrointestinal side effects that undermine long-term adherence (Song et al., 2022). Likewise, although anabolic drugs such as teriparatide can promote bone formation, their broad use is constrained by complicated dosing regimens, substantial costs, and possible safety concerns (Yuan et al., 2023). Consequently, it is urgently important to further elucidate the molecular pathophysiology underlying osteoporosis and to discover new therapeutic targets, especially interventions aimed at selectively strengthening osteoblast function.

Recent evidence indicates that, in addition to traditional endocrine influences and aging-associated drivers (Jinteng et al., 2023), cellular stress responses and distinct programmed cell death mechanisms are deeply involved in the development of osteoporosis (Chen et al., 2025; Chen C. et al., 2024; Li et al., 2024a; Zhang P. et al., 2024; Chen T. et al., 2024; Wu et al., 2025; Su et al., 2023). In this context, disulfidptosis—a newly described death program triggered by disulfide-bond stress—has attracted increasing interest because it is tightly linked to dysregulated cysteine metabolism and cytoskeletal injury (Zhang P. et al., 2024; Wang et al., 2024). In the bone marrow microenvironment, bone marrow mesenchymal stem cells (BM-MSCs) constitute a key foundational cell population with multilineage differentiation potential and are essential for sustaining bone formation (Li et al., 2024b). However, under osteoporotic conditions, BM-MSC lineage commitment becomes disturbed, typically presenting as weakened osteogenic differentiation alongside an enhanced propensity for adipogenic differentiation, which promotes marrow adiposity and contributes to reduced bone mass (Song et al., 2022; Zhang Z. et al., 2024). Accordingly, dysfunction of BM-MSCs is a major element of osteoporosis pathophysiology. Although prior work has suggested that disulfide-associated proteins can participate in regulating bone metabolic homeostasis in osteoporosis (Lu et al., 2025), the detailed regulatory basis and pathological relevance of disulfidptosis in osteoporosis—particularly within the osteogenic lineage—have not yet been adequately clarified (Zhang P. et al., 2024).

**FIGURE 1:**
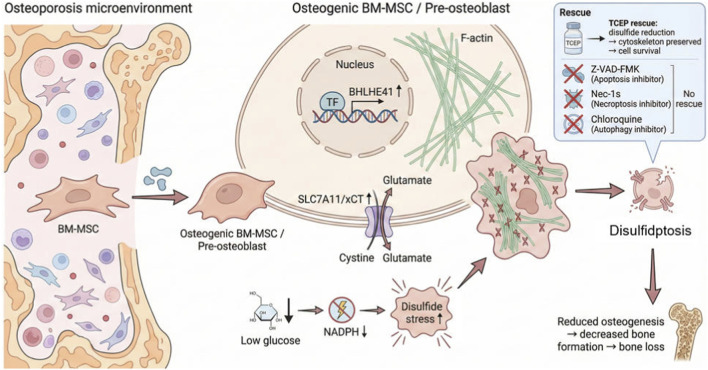
Mechanistic schematic of disulfidptosis-associated osteogenic impairment in osteoporosis.

In this work, we seek to comprehensively define the cell type–dependent involvement of disulfidptosis within the osteoporotic bone marrow microenvironment, with a particular focus on its relationship to BM-MSC osteogenic differentiation, and to pinpoint pivotal regulatory nodes, thereby offering a new theoretical framework for interventions aimed at correcting bone metabolic imbalance. A mechanistic schematic illustrating how disulfidptosis contributes to osteoporosis is shown in Figure 1.

## 2 Methods

### 2.1 Microarray data acquisition and processing

The gene expression profile was retrieved from the Gene Expression Omnibus (GEO) database under accession GSE56815 (Barrett et al, 2013). This dataset relies on the Affymetrix platform and includes monocytes from 20 osteoporosis (OP) samples and 20 normal control (NC) samples. The raw data were processed using the ‘oligo’ package in R. Background correction and normalization were performed using the Robust Multi-array Average (RMA) algorithm. The expression matrix was log2-transformed, and probes were annotated to gene symbols for downstream analysis.

### 2.2 Weighted gene Co-expression network analysis (WGCNA)

To screen for gene co-expression modules linked to osteoporosis (OP), we conducted WGCNA using the “WGCNA” package in R. A soft-thresholding parameter of β = 6 was chosen according to the scale-free topology fitting results together with mean connectivity evaluation, and a signed network was then constructed. Genes were hierarchically clustered based on a topological overlap matrix (TOM)–derived dissimilarity measure to generate gene dendrograms, and modules were subsequently detected using the dynamic tree cut procedure, with the minimum module size set to 30. Module eigengenes (MEs) were computed as representative expression signatures for each module. To determine clinical relevance, ME values were correlated with sample traits (OP vs. NC), and the resulting correlation coefficients and corresponding P-values were displayed in heatmaps. Modules satisfying the significance criteria (|correlation| > 0.5 and P < 0.05) were selected for downstream analyses.

### 2.3 Differential expression analysis and gene set intersection

Differential expression between the osteoporosis (OP) group and normal controls (NC) was assessed with the ‘limma’ package. Genes were defined as differentially expressed when they met the criteria of |log2 fold change| > 1 and adjusted P-value <0.05. The overall distribution of significantly upregulated (red) and downregulated (blue) genes was displayed using a volcano plot. A set of 52 disulfidptosis-related genes was compiled from published literature, and Venn diagram overlap analysis was performed between this gene set and genes contained in the OP-associated WGCNA module (e.g., the turquoise module) to obtain candidate disulfidptosis-related genes. The expression patterns of these candidates were further examined using boxplots, and statistical differences between groups were tested by the Wilcoxon method, with P < 0.05 considered significant.

### 2.4 Consensus clustering and principal component analysis

To stratify samples according to the expression patterns of candidate disulfidptosis-related genes, consensus clustering was carried out in R using the “ConsensusClusterPlus” package. The most suitable cluster number (k) was selected by jointly examining the cumulative distribution function (CDF) curves and the consensus matrix heatmaps across k values from 2 to 9. Sample membership across clustering solutions was further summarized using a tracking plot to show classification stability. In parallel, principal component analysis (PCA) was performed with the ‘prcomp’ function to depict the distribution of samples between the identified clusters, and the resulting PCA figures were generated with the “ggplot2” package.

### 2.5 Single-cell RNA sequencing data analysis

Single-cell RNA sequencing (scRNA-seq) data from relevant cohorts (e.g., publicly available datasets) were analyzed using the ‘Seurat’ package (version 4.0). For quality control, cells were retained only if they contained 200–5,000 detected genes and exhibited a mitochondrial gene proportion of <20%. The data were then normalized and scaled, and dimensionality reduction was conducted using SCTransform followed by principal component analysis (PCA); the resulting low-dimensional embeddings were visualized with uniform manifold approximation and projection (UMAP). Cell type annotation was performed based on canonical marker genes curated from the CellMarker database. To quantify disulfidptosis score at the single-cell level, disulfidptosis scores were computed for each cell using the “AUCell” package with the disulfidptosis-related gene set, and score differences between OP and control groups were evaluated using the Wilcoxon test.

### 2.6 BM-MSC subpopulation analysis

Bone marrow mesenchymal stem cells (BM-MSCs) were extracted from the scRNA-seq dataset and subjected to re-clustering to resolve finer BM-MSC subpopulations (e.g., Chondrogenic, Osteogenic, Terminal1, and Terminal2). The expression patterns of representative marker genes for each subcluster were displayed using dot plots. Disulfidptosis scores were then evaluated across BM-MSC subpopulations and further compared between the OP and control groups; these differences were visualized with violin plots and tested for significance using the Wilcoxon method.

### 2.7 SCENIC analysis for transcription factor regulon inference and chromatin remodeling signature scoring

To infer transcription factor–centered regulatory programs at the single-cell level, single-cell regulatory network inference and clustering (SCENIC) was carried out in R using the “SCENIC” package to identify transcription factor (TF) regulons. TF activity patterns were summarized and displayed as heatmaps, and selected TFs (e.g., BHLHE41) were further examined for expression differences between groups. In addition, regulatory network diagrams were constructed to depict TF–target relationships, such as the interactions of BHLHE41 with SLC7A11 and BCL2.

To characterize chromatin remodeling–related transcriptional programs in osteoblasts, curated gene signatures capturing ATP-dependent remodeling ATPases, epigenetic activators (chromatin opening/transcriptional activation), and epigenetic repressors (chromatin closing/transcriptional silencing) were scored for each cell using Seurat AddModuleScore. Signature scores were compared between NC and OP osteoblasts using a two-sided Wilcoxon rank-sum test. Osteoblasts were further stratified into BHLHE41-high and BHLHE41-low groups (median split), and representative chromatin regulators (EHMT2, HDAC1, and SIRT1) were compared between groups.

### 2.8 Statistical analysis

All statistical analyses were performed in R (version 4.2.0). Unless otherwise stated, data are expressed as mean ± standard error. For comparisons of continuous variables between groups, Student’s t-test or the Wilcoxon rank-sum test was applied as appropriate. Correlation assessments were carried out using Pearson or Spearman correlation methods. A P-value <0.05 was regarded as statistically significant, and significance levels are indicated as *P < 0.05, **P < 0.01, and ***P < 0.001.

## 3 Results

### 3.1 Systematic identification of core genes associated with disulfidptosis in osteoporosis

**FIGURE 2:**
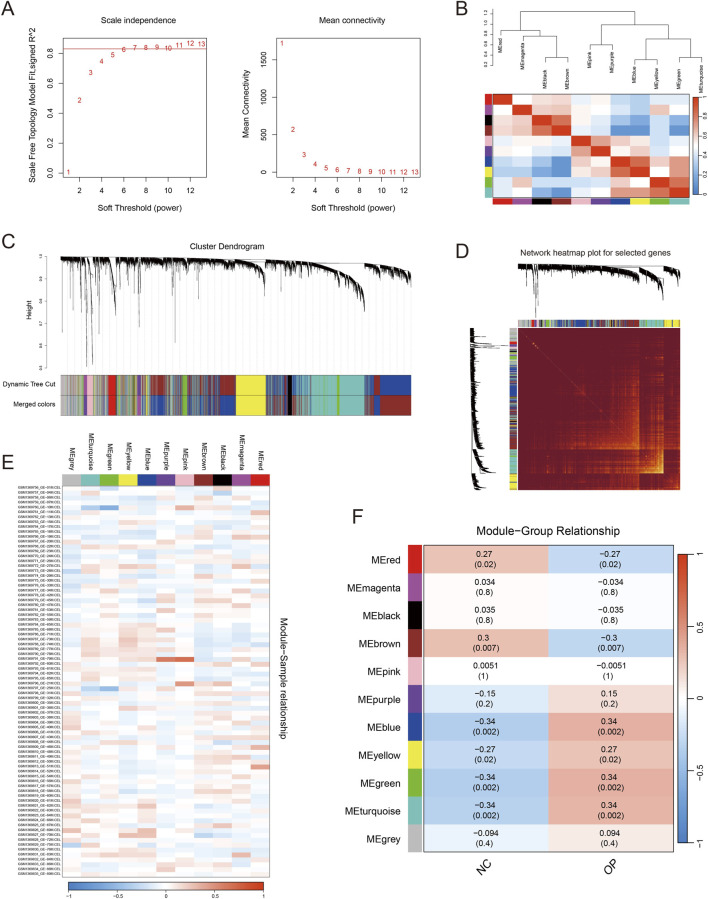
Weighted gene co-expression network analysis (WGCNA) reveals gene modules associated with osteoporosis. **(A)** Scale-free topology model fit (left) and mean connectivity (right) across candidate soft-thresholding powers; β = 6 was chosen. **(B)** Dendrogram clustering of module eigengenes accompanied by a heatmap summarizing correlations among modules. **(C)** Gene hierarchical clustering dendrogram constructed using topological overlap–based dissimilarity; colored bars denote module assignments identified by Dynamic Tree Cut and the corresponding merged modules (Merged colors). **(D)** Topological overlap matrix (TOM) heatmap illustrating network connectivity patterns among the selected genes. **(E)** Heatmap displaying module eigengene expression patterns across all samples. **(F)** Heatmap of module–trait relationships, where each cell reports the correlation coefficient and the corresponding P-value (in parentheses) between module eigengenes and clinical traits (NC: Normal Control; OP: Osteoporosis); red indicates positive correlation and blue indicates negative correlation.

To comprehensively screen osteoporosis (OP)–related genes, we applied weighted gene co-expression network analysis (WGCNA) to the bulk monocyte microarray expression data from GSE56815. The parameters required for network construction were optimized by soft-threshold power selection (Figure 2A). Using this framework, we successfully pinpointed key gene modules that displayed significant associations with the OP phenotype (Figures 2B,C). We then generated a co-expression network heatmap to visualize the overall connectivity structure among genes (Figure 2D). The results revealed strong co-expression patterns within the candidate gene set, and most of these genes were concentrated in the MEturquoise module. Moreover, additional evaluation demonstrated that the characteristic expression signature of the MEturquoise module showed broad dysregulation in osteoporosis samples (Figures 2E,F). Collectively, these observations suggest that the MEturquoise module constitutes a gene cluster tightly linked to osteoporosis pathogenesis, thereby supporting our decision to prioritize this module in subsequent analyses.

**FIGURE 3:**
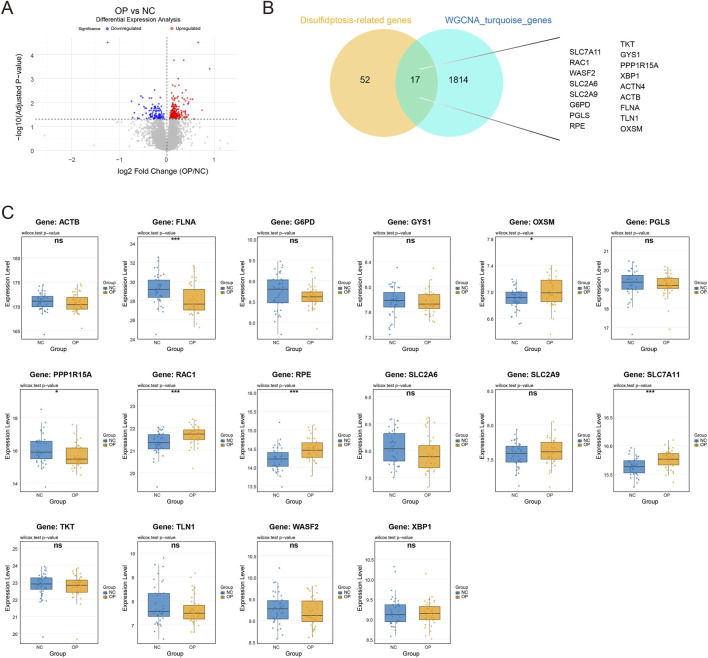
Identification of disulfidptosis-related hub genes in osteoporosis. **(A)** Volcano plot displaying differentially expressed genes (DEGs) between osteoporosis (OP) and normal control (NC) samples; red points indicate upregulated genes and blue points indicate downregulated genes. **(B)** Venn diagram depicting the overlap between the disulfidptosis-related gene set (52 genes) and genes from the WGCNA turquoise module (1814 genes), yielding 17 shared candidate genes. **(C)** Boxplots showing validation of the expression levels of the 17 candidate genes in the bulk RNA-seq dataset. Statistical significance was evaluated using the Wilcoxon test (P < 0.05, *P < 0.01, **P < 0.001, ns: not significant). Notably, SLC7A11 is markedly upregulated in the OP group.

To pinpoint core genes within this module that are linked to the disulfidptosis pathway in osteoporosis, we first carried out differential expression analysis. Relative to the normal control (NC) group, the osteoporosis (OP) group displayed a large number of significantly differentially expressed genes (Figure 3A). Next, we overlapped the curated disulfidptosis-related gene set with genes contained in the MEturquoise module, which yielded 17 core candidate genes, including representative regulators such as SLC7A11 and RAC1 (Figure 3B). We then evaluated whether these candidates showed genuine expression shifts in OP by comparing their transcript levels between the NC and OP cohorts. As illustrated in Figure 3C, several candidate genes differed significantly across groups, and SLC7A11 in particular exhibited an extremely significant increase in the osteoporosis group. Moreover, genes related to cytoskeletal organization (e.g., FLNA, MYH9) as well as genes implicated in mitochondrial function (e.g., NDUFS1, LRPPRC) also showed marked expression alterations. Together, these results provide transcriptional evidence that disulfidptosis-associated genes are broadly dysregulated in the osteoporotic pathological milieu, and they further imply that SLC7A11 may act as a central hub gene contributing to bone metabolic imbalance.

### 3.2 Molecular subtyping of osteoporosis reveals disease heterogeneity

**FIGURE 4:**
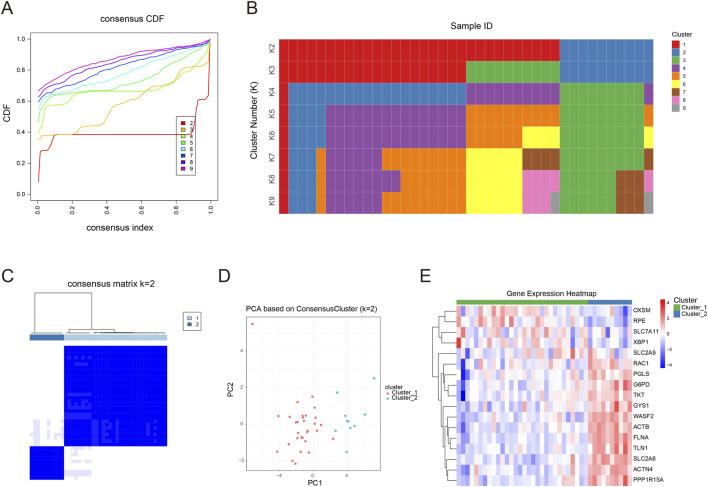
Disulfidptosis-related molecular subtyping of osteoporosis samples. **(A)** Cumulative distribution function (CDF) curves of consensus clustering coefficients across candidate cluster numbers (k = 2–9). **(B)** Consensus clustering tracking plot illustrating how sample assignments change under different k settings. **(C)** Consensus matrix heatmaps for k = 2, showing clear separation between the two clusters. **(D)** Principal component analysis (PCA) plot depicting the distribution of samples according to the two identified clusters. **(E)** Heatmap displaying the expression patterns of the 17 disulfidptosis-related genes across the two clusters.

To investigate osteoporosis heterogeneity and assess whether distinct disulfidptosis-related molecular patterns could be distinguished, we conducted consensus clustering using the expression signatures of the 17 core genes. By evaluating clustering robustness across different k values with cumulative distribution function (CDF) curves and consensus matrix heatmaps, k = 2 was selected as the most stable and appropriate solution (Figures 4A,B), thereby classifying all samples into two molecular subtypes, termed Cluster_1 and Cluster_2. Under this setting, the consensus matrix, together with principal component analysis, demonstrated that samples could be separated clearly and consistently into two groups, showing strong within-cluster similarity and marked between-cluster differences (Figures 4C,D). We additionally constructed a heatmap to display the expression landscape of the key genes (Figure 4E), which revealed broadly divergent expression patterns between the two subtypes. Co-expression–based gene grouping further indicated several gene modules: OXSM, RPE, SLC7A11, G6PD, and TKT were notably upregulated in Cluster_1, whereas WASF2, ACTB, FLNA, TLN1, ACTN4, and PPP1R15A showed higher expression in Cluster_2. Collectively, these molecular findings provide clear evidence of pronounced heterogeneity within osteoporosis.

### 3.3 Single-cell transcriptomics reveals bone marrow microenvironment remodeling and disulfidptosis in osteoporosis

**FIGURE 5:**
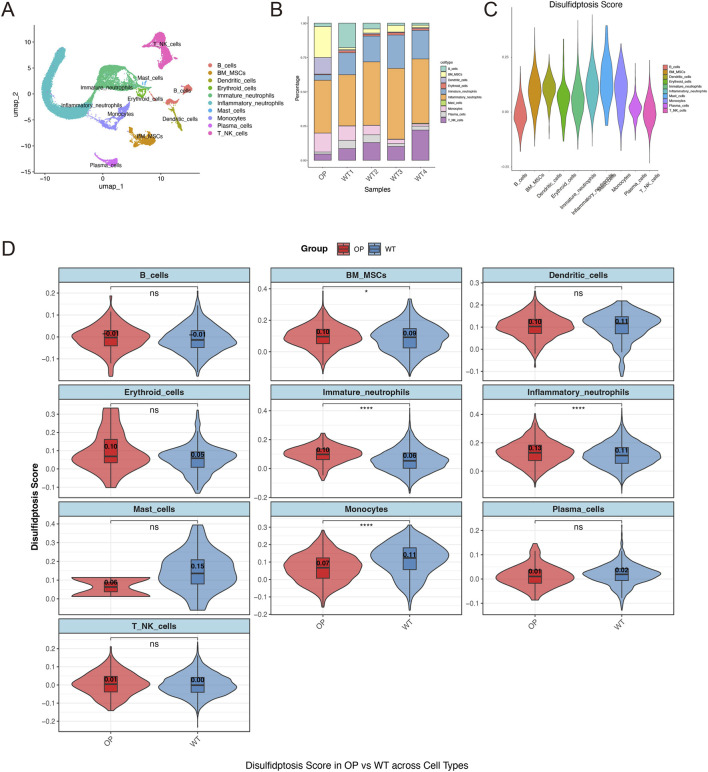
Single-cell atlas of the bone marrow microenvironment and disulfidptosis features in osteoporosis. **(A)** UMAP visualization of the principal cell populations annotated in the single-cell dataset. **(B)** Stacked bar plot summarizing the relative abundance of each cell type across individual samples. **(C)** Violin plot illustrating the distribution of disulfidptosis scores among different cell types. **(D)** Within each cell type, disulfidptosis scores were compared between OP and NC groups; significance was assessed using the Wilcoxon test (P < 0.05, ***P < 0.0001). BM-MSCs and neutrophil populations show significantly elevated scores in the OP group.

To characterize the disease microenvironment with higher resolution, we conducted single-cell RNA sequencing analysis. UMAP projection generated a detailed bone marrow cellular atlas in which cells were clearly separated into multiple distinct clusters (Figure 5A), encompassing bone marrow mesenchymal stem cells, osteoblasts, B cells, T cells, myeloid populations, and other cell types. Quantification of cell-type proportions indicated that the bone marrow microenvironment is reshaped in osteoporosis, showing an increased fraction of BM-MSCs together with relative reductions in most immune cell populations (Figure 5B). We next estimated “disulfidptosis signature” using computational scoring, which revealed pronounced cell-type heterogeneity; notably, BM-MSCs displayed significantly higher scores than other cell groups (Figure 5C). Further comparisons between groups (Figure 5D) showed that immature neutrophils and inflammatory neutrophils had extremely significant score elevations in the OP group (p < 0.001), and BM-MSCs likewise exhibited significantly higher scores relative to the NC control group (p < 0.01). By contrast, B cells, dendritic cells, T-NK cells, and mast cells did not show significant score differences between groups, whereas monocytes presented a significant reduction in the OP group (p < 0.05). Together, these results suggest that osteoporosis selectively reprograms the disulfidptosis state in specific cellular subsets within the bone marrow niche.

### 3.4 The osteogenic subpopulation of BM-MSCs is a specific target of disulfidptosis stress

**FIGURE 6:**
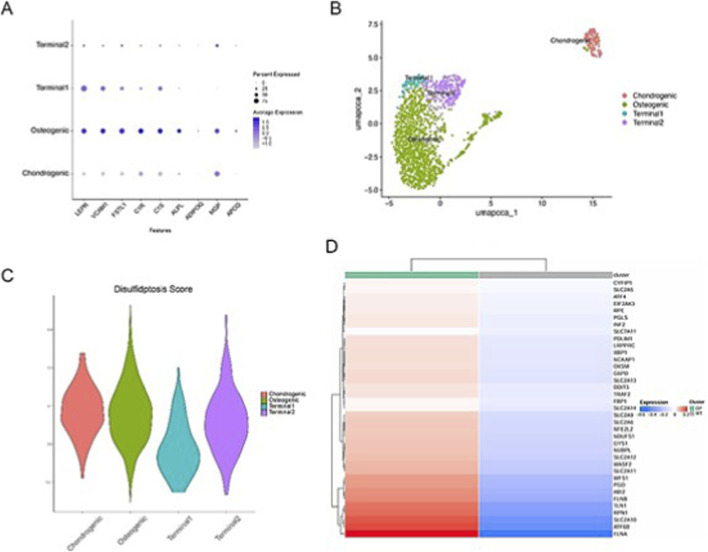
Disulfidptosis characterization across BM-MSC subpopulations. **(A)** Dot plot summarizing the expression of marker genes used to define BM-MSC subclusters (Chondrogenic, Osteogenic, Terminal1, Terminal2). **(B)** UMAP visualization showing re-clustering results for BM-MSCs. **(C)** Violin plot depicting the distribution of disulfidptosis scores across the four BM-MSC subpopulations. **(D)** Heatmap comparing the expression of disulfidptosis-related genes within the osteogenic/osteoblast-associated subpopulation between OP and NC groups.

Because BM-MSCs appeared to be the main cell population exhibiting disulfidptosis signals and also showed substantial intrapopulation heterogeneity, we further carried out subclustering analysis. By integrating UMAP visualization with marker-gene dot plots, BM-MSCs were clearly partitioned into four subsets: an osteogenic subset, a chondrogenic subset, and two terminally differentiated subsets. These four subpopulations displayed distinct differentiation trajectories and characteristic transcriptional programs (Figures 6A,B). Building on this classification, we next evaluated disulfidptosis score across the BM-MSC subsets. The results delineated the baseline distribution of Disulfidptosis Scores among these BM-MSC subpopulations (Figure 6C). Moreover, within the osteogenic subset, disulfidptosis-related genes were broadly expressed at higher levels in the osteoporosis group compared with the normal control group (Figure 6D). Together, these findings indicate that, under osteoporotic conditions, BM-MSCs show a selective increase in disulfidptosis susceptibility within cell subpopulations associated with osteogenic differentiation. Notably, while the overall disulfidptosis score varied, the key regulator SLC7A11 remained consistently upregulated, suggesting a specific adaptive transcriptional response in this lineage.

### 3.5 Transcription factor BHLHE41 mediates disulfidptosis in osteoporotic BM-MSCs by regulating SLC7A11

**FIGURE 7:**
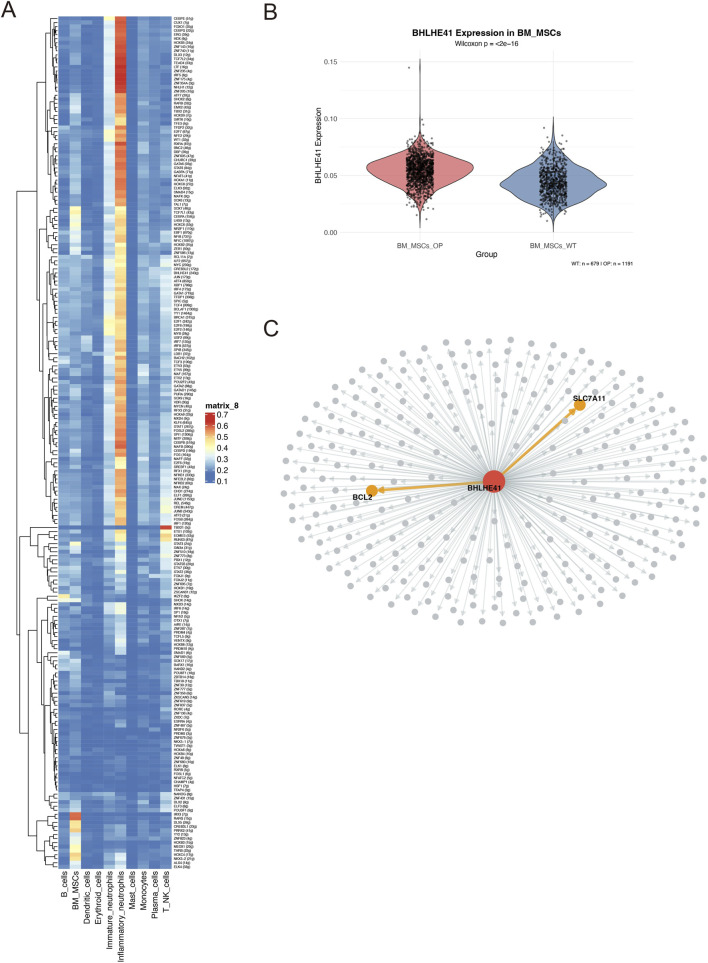
Identification of the BHLHE41/SLC7A11 regulatory axis in BM-MSCs. **(A)** Heatmap summarizing transcription factor (TF) regulon activity inferred by SCENIC analysis. **(B)** Violin plot comparing BHLHE41 expression in BM-MSCs, showing a significant increase in the OP group relative to NC (P < 2e-16). **(C)** BHLHE41-centered transcriptional regulatory network. Orange arrows denote predicted regulatory links from BHLHE41 to the target genes SLC7A11 and BCL2.

Prior analyses showed that SLC7A11 is markedly upregulated in osteoporotic BM-MSCs. To clarify the upstream regulatory basis for this change, we conducted transcription factor enrichment analysis. The results suggested that BHLHE41 is a candidate upstream regulator that is significantly increased in OP BM-MSCs and shows the strongest correlation with SLC7A11 expression (Figure 7A). Single-cell–level validation further demonstrated that BHLHE41 expression in osteoporotic BM-MSCs was extremely significantly higher than that in controls (Wilcoxon p < 2e-16) (Figure 7B). We then performed co-expression analysis and regulatory network construction, which yielded a BHLHE41-centered network that included SLC7A11 as well as the apoptosis-associated gene BCL2 (Figure 7C). Notably, this regulatory framework not only directly connected to SLC7A11 but also incorporated BCL2, a gene closely linked to apoptotic regulation. Taken together, these data support the conclusion that the transcription factor BHLHE41 acts as a critical upstream driver of SLC7A11 upregulation in osteoporosis.

### 3.6 Chromatin remodeling programs are associated with BHLHE41 expression in osteoblasts

**FIGURE 8:**
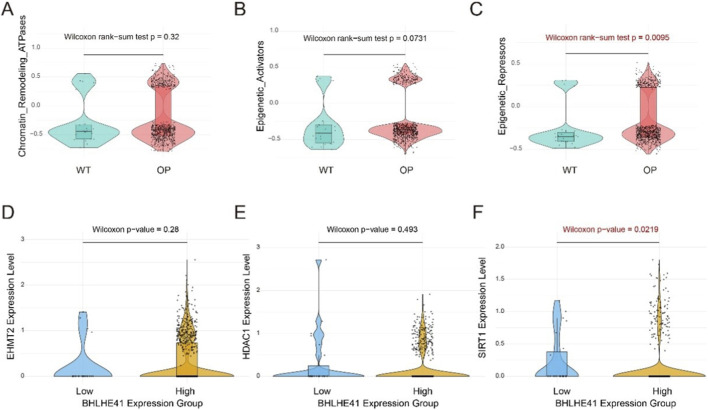
BHLHE41 may influence the chromatin remodeling state of osteoblast subpopulations via SIRT1. **(A–C)** Comparison of chromatin remodeling–related pathway activity scores in osteoblast subpopulations between wild-type (NC) and osteoporosis (OP) samples, including ATP-dependent chromatin remodeling activity (Chromatin_Remodeling_ATPases), an epigenetic activator signature (Epigenetic_Activators), and an epigenetic repressor signature (Epigenetic_Repressors). **(D–F)** Expression of EHMT2, HDAC1, and SIRT1 in osteoblasts stratified by BHLHE41 expression (Low vs. High). Each dot represents a single cell. Violin plots show the distribution, and embedded boxplots indicate the median and interquartile range. Statistical significance was assessed using a two-sided Wilcoxon rank-sum test; P values are shown in the panels.

Given the transcriptional repressor identity of BHLHE41, we next assessed whether osteoblast subpopulations exhibit altered chromatin remodeling programs in osteoporosis (OP). Module scoring showed that ATP-dependent chromatin remodeling activity was comparable between NC and OP osteoblasts (Chromatin_Remodeling_ATPases; Wilcoxon rank-sum test, *p* = 0.32; Figure 8A), while the epigenetic activator signature displayed a non-significant upward trend in OP (Epigenetic_Activators; *p* = 0.0731; Figure 8B). Notably, the epigenetic repressor signature—reflecting chromatin closing and transcriptional silencing—was significantly elevated in OP osteoblasts (Epigenetic_Repressors; *p* = 0.0095; Figure 8C), indicating a shift toward a more repressive chromatin state. To link this phenotype to BHLHE41, we stratified osteoblasts by BHLHE41 expression (Low vs. High) and examined representative chromatin regulators. Among EHMT2, HDAC1, and SIRT1, only SIRT1 was significantly higher in the BHLHE41-high group (Wilcoxon test, *p* = 0.0219), whereas EHMT2 and HDAC1 did not differ between groups (*p* = 0.28 and 0.493, respectively; Figures 8D–F). Together, these results support an association between elevated BHLHE41 and a SIRT1-linked chromatin repression program in osteoblasts, providing a mechanistic context for downstream stress-response phenotypes.

## 4 Discussion

In this work, we integrated bulk transcriptomic microarray profiles of circulating monocytes with bone marrow single-cell RNA sequencing (scRNA-seq) data and, together with multiple machine learning approaches and molecular network–based analyses, systematically uncovered regulatory programs that shape cellular responses to disulfide-bond stress in osteoporosis. Our results highlight SLC7A11 as a central hub gene involved in disulfidptosis during osteoporosis. At single-cell resolution, we further show that BM-MSCs—especially the subpopulations committed to osteogenic differentiation—represent the major cellular targets of disulfidptosis-related stress, with broadly increased expression of disulfidptosis-associated genes in the osteogenic BM-MSC subset in osteoporosis (Figure 6D). In addition, mechanistic analyses identified the transcription factor BHLHE41 as a critical upstream regulator that controls SLC7A11 expression and modulates disulfidptosis in BM-MSCs. Beyond transcriptional regulation, module scoring in osteoblasts suggested altered epigenetic state programs in osteoporosis, and stratification by BHLHE41 expression highlighted SIRT1 as a candidate epigenetic effector within a repressive chromatin-state program.

The pathophysiological basis of osteoporosis is multifaceted. In addition to the traditional view centered on an imbalance between bone resorption and bone formation, accumulating evidence indicates that endoplasmic reticulum stress, mitochondrial impairment, and multiple regulated cell death programs also participate in disease progression. Disulfidptosis is a recently described form of regulated cell death initiated by disulfide-bond stress. Mechanistically, it is characterized by abnormal intracellular accumulation of disulfides (e.g., cysteine), which drives excessive disulfide bond formation among actin cytoskeletal proteins, ultimately resulting in collapse of the cytoskeletal network and subsequent cell death (Wang L. et al., 2024). Using WGCNA together with differential expression analysis, our study identified several disulfidptosis-related genes—including SLC7A11—that are significantly dysregulated in osteoporosis.

SLC7A11 (solute carrier family 7 member 11), also termed xCT, functions as the light-chain component of the cystine–glutamate antiporter system Xc^−^ and is essential for preserving intracellular redox homeostasis (Miao et al., 2021). In our analysis, SLC7A11 expression was increased in osteoporosis; however, the disulfidptosis score within the osteogenic differentiation subpopulation was decreased. This pattern differs from findings in oncology studies, where elevated SLC7A11 is reported to facilitate disulfidptosis (Wang Z. et al., 2024). Such an inconsistency may reflect the combined influence of cell-type specificity and microenvironmental metabolic context. Indeed, how disulfidptosis interfaces with redox regulation can vary substantially across physiological and pathological settings (Liu et al., 2024). In glucose-depleted tumor microenvironments, high metabolic demand together with strong SLC7A11 activity can drive rapid NADPH consumption, thereby predisposing cells to disulfidptosis (Koppula et al., 2021; Liu et al., 2020). By contrast, within the osteoporotic bone marrow niche, although metabolic stress and persistent inflammation are present, the local environment may not reach the degree of glucose scarcity required to robustly activate full-scale disulfidptosis (Liu et al., 2023; Zheng et al., 2020). Under these conditions, maintaining SLC7A11 at a certain baseline may instead favor cystine import and glutathione production, strengthening antioxidant capacity and thereby restraining disulfidptosis. This compensatory pattern may be especially evident in osteogenic differentiation subpopulations, supporting their preferential survival under microenvironmental stress.

At the same time, osteogenic differentiation is an energetically demanding process that relies on sustained cytoskeletal remodeling and adequate energy supply (Yan et al., 2023), and marked energy insufficiency can directly suppress this differentiation potential. Notably, the SLC7A11-driven cystine metabolic pathway is itself highly energy-consuming (He et al., 2022). Thus, in the osteoporotic microenvironment, BM-MSCs may prioritize energy expenditure toward redox maintenance, leaving inadequate energetic resources to support osteogenic differentiation. Moreover, in an inflammatory milieu, lipid metabolites generated under oxidative stress can inhibit BM-MSC differentiation toward osteoblasts while shifting lineage commitment toward adipogenesis (Parhami et al., 2000; Van Gastel and Carmeliet, 2021; Rendina-Ruedy and Rosen, 2020). The abnormal expansion and accumulation of adipocytes in osteoporotic bone marrow (Li et al., 2020) can further impair mitochondrial function in surrounding cells via secretion of factors such as fatty acids (Singh et al., 2017), competitively reduce local glucose availability (Chaves Neto et al., 2018), and reshape substrate utilization within the microenvironment, thereby promoting chronic inflammation (King et al., 2022; Ning et al., 2022). Together, these changes intensify metabolic stress and further impede osteoblastic differentiation.

At the mechanistic level, our results highlight for the first time the central involvement of the BHLHE41–SLC7A11 axis in osteoporosis. BHLHE41 is a nuclear transcriptional repressor within the basic helix–loop–helix (bHLH) protein superfamily and has been implicated in a range of physiological functions as well as tumorigenesis (Bret et al., 2025; Azmi et al., 2003). Recent work has also suggested a role for BHLHE41 in osteoclast differentiation (Zhang Y. et al., 2024). In contrast, our study is the first to identify its key regulatory contribution to disulfidptosis within osteogenic-lineage cells. We observed that increased BHLHE41 expression is strongly associated with elevated SLC7A11 expression, supporting the possibility that BHLHE41 acts as a protective regulator that sustains SLC7A11 levels to preserve cellular redox balance (Zheng et al., 2018). A moderate activation state of this axis may enable cells to withstand disulfidptosis under metabolic stress, while potentially promoting redistribution of cellular energy resources, thereby indirectly modulating osteogenic differentiation capacity. Notably, BHLHE41 is a transcriptional repressor, which is conceptually consistent with the elevated chromatin repression signature observed in OP osteoblasts. SIRT1, a NAD + -dependent deacetylase with established roles in chromatin regulation, may provide a mechanistic bridge linking metabolic stress to epigenetic state transitions. The preferential increase of SIRT1 in BHLHE41-high osteoblasts raises the possibility that BHLHE41 may cooperate with, or converge on, SIRT1-associated deacetylation programs to reinforce a transcriptionally repressive chromatin environment. Future studies should test whether BHLHE41 directly regulates SIRT1, and whether perturbing SIRT1 alters chromatin accessibility and stress-response gene programs relevant to disulfide stress susceptibility in osteogenic-lineage cells.

Although this study provides important insights, several limitations should be acknowledged. First, our conclusions are derived largely from bioinformatics pipelines and machine learning–assisted analyses; therefore, the causal contribution of the BHLHE41/SLC7A11 axis to osteoporosis *in vivo* still needs to be confirmed, ideally using conditional knockout or overexpression strategies in relevant animal models. Second, the chromatin remodeling signals reported here were inferred from transcriptome-derived module scores rather than direct epigenomic measurements. Orthogonal validation using epigenomic profiling (e.g., scATAC-seq and/or ChIP-/CUT and Tag-based assays for histone acetylation/methylation marks) and functional perturbation of SIRT1 will be required to confirm true chromatin-state changes and to establish a causal BHLHE41–SIRT1 epigenetic axis in osteoblast subpopulations. Third, while we observed that osteogenic differentiation–associated subpopulations of bone marrow mesenchymal stem cells exhibit relative resistance to disulfidptosis, the precise impact of disulfidptosis on BM-MSC osteogenic differentiation, as well as the downstream molecular events, remains insufficiently defined. For example, at glucose levels that permit disulfidptosis induction, the specific actin cytoskeletal proteins that undergo disulfide modification—and the functional consequences of these modifications—require more detailed characterization. Future studies should therefore validate this pathway in more complex *in vivo* systems, investigate upstream signals that control BHLHE41 expression, and screen small-molecule candidates capable of balancing cell survival with osteogenic capacity, thereby supporting precision therapeutic development for osteoporosis. Although this study provides important insights, several limitations should be acknowledged. First, our conclusions are derived largely from bioinformatics pipelines and machine learning–assisted analyses; therefore, the causal contribution of the BHLHE41/SLC7A11 axis to osteoporosis *in vivo* still needs to be confirmed, ideally using conditional knockout or overexpression strategies in relevant animal models. Second, the chromatin remodeling signals reported here were inferred from transcriptome-derived module scores rather than direct epigenomic measurements. Orthogonal validation using epigenomic profiling (e.g., scATAC-seq and/or ChIP-/CUT and Tag-based assays for histone acetylation/methylation marks) and functional perturbation of SIRT1 will be required to confirm true chromatin-state changes and to establish a causal BHLHE41–SIRT1 epigenetic axis in osteoblast subpopulations. Third, while we observed dysregulated disulfidptosis-related programs in osteogenic-lineage BM-MSC subpopulations, the precise impact of disulfidptosis on BM-MSC osteogenic differentiation, as well as downstream molecular events (including potential actin disulfide modifications), remains insufficiently defined and warrants further investigation.

## 5 Conclusion

In conclusion, our study delineates cell type–dependent mechanisms underlying disulfidptosis dysregulation in osteoporosis. The findings emphasize that relieving metabolic limitations within the bone marrow niche—and ensuring adequate energy support—may be crucial for improving osteoporosis outcomes. In addition, we propose the BHLHE41–SLC7A11 axis as a key pathway that connects transcriptional control with bone metabolic imbalance. Furthermore, transcriptome-based chromatin remodeling signature scoring in osteoblasts suggests that BHLHE41 is associated with a repressive chromatin-state program, with SIRT1 emerging as a candidate epigenetic effector. Taken together, these results establish a conceptual basis for precision therapeutic strategies aimed at targeting disulfide-stress vulnerability in osteoporosis.

## Data Availability

The original contributions presented in the study are included in the article/supplementary material, further inquiries can be directed to the corresponding authors.
